# Imaging in Oncology

**DOI:** 10.1038/sj.bjc.6602766

**Published:** 2005-10-11

**Authors:** J Hague, M A Hall-Craggs

**Affiliations:** 1 University College Hospital, London, UK

This second edition of this two-volume text follows on from the first edition published in 1998, itself the winner of the Royal Society of Medicine's Multi-Author textbook of the year. This volume was written during 2003 by leaders in their fields, mainly from the UK with contributions from Europe and the USA. Radiologists comprise the majority of the authors (82 out the 91 contributors), although there is substantial input from clinical oncologists, as the editors have tried to produce a text that is clinically relevant and interesting to read.

The first volume begins with informative and readable chapters on imaging strategies, staging methods, assessment of tumour response to therapy and second malignancies. There is a chapter outlining the principles of treatment, which is particularly useful to the radiologist as these areas are often outside the scope of imaging training.

Expanded chapters on the common malignancies follow, covering malignancies frequently seen in the day-to-day practice of a general imaging department. There are some inevitable omissions for a two-volume text: for example, penile cancer is not covered, and there is limited coverage of many rarer tumours (for example, some sarcomas).

The new edition of this text keeps the essential structure and format of the original but there are several useful new additions, including chapters on adrenal and splenic tumours. Also of particular interest is a section on horizons in imaging, outlining MR lymphography, imaging angiogenesis, and molecular imaging.

All chapters have been updated, some rewritten with new authors, for example paediatric tumours, and an excellent new chapter on lung and pleural metastases. The chapters are generally well referenced; the most recent references given are from 2002.

The great strength of this text is the simplicity and clarity of presentation. The text is supplemented by very clear, uncluttered diagrams enabling the staging of tumours to be easily understood. For radiologists with visual memories, the diagrammatic depiction of staging is much easier to appreciate and remember than long lists. The text is also supplemented by useful colour boxes, some giving key points that make quick reference straightforward. The images are large and well selected, with an emphasis heavily towards CT and MR – although there are some good examples of pathological correlates. PET/CT PET is briefly touched upon, where relevant.

The text is essentially directed at a radiological audience and is particularly appropriate for general radiology departments and trainee radiologists. It has a less obvious role for the specialist radiologist. The text would be of interest to oncologists, particularly those in training, to supplement their understanding and knowledge of the imaging appearance of cancers. It would be a valuable addition to any oncology department library.

